# *Cdc42* deletion yielded enamel defects by disrupting mitochondria and producing reactive oxygen species in dental epithelium

**DOI:** 10.1016/j.gendis.2023.101194

**Published:** 2023-12-12

**Authors:** Jinxuan Zheng, Rongcheng Yu, Yiqi Tang, Sihui Su, Sainan Wang, Chenxi Liao, Xuecong Li, Jiabin Liao, Dongsheng Yu, Tingting Ai, Wei Zhao, Vicky Yau, Chufeng Liu, Liping Wu, Yang Cao

**Affiliations:** a Hospital of Stomatology, Guanghua School of Stomatology, Guangdong Provincial Key Laboratory of Stomatology, Sun Yat-sen University, Guangzhou, Guangdong 510055, China; b Guangdong Provincial Key Laboratory of Oral Diseases, Guangzhou, Guangdong 510055, China; c Department of Cariology and Endodontology, Peking University School and Hospital of Stomatology & National Center for Stomatology & National Clinical Research Center for Oral Diseases & National Engineering Research Center of Oral Biomaterials and Digital Medical Devices, Beijing 100081, China; d Department of Oral and Maxillofacial Surgery, University at Buffalo, Buffalo, NY 14214, USA; e Department of Orthodontics, Stomatological Hospital, Southern Medical University, Guangzhou, Guangdong 510280, China

**Keywords:** Cdc42, Enamel defects, Mitochondrial dysfunction, Rho GTPase, Tooth repair

## Abstract

Developmental defects of enamel are common due to genetic and environmental factors before and after birth. Cdc42, a Rho family small GTPase, regulates prenatal tooth development in mice. However, its role in postnatal tooth development, especially enamel formation, remains elusive. Here, we investigated Cdc42 functions in mouse enamel development and tooth repair after birth. Cdc42 showed highly dynamic temporospatial patterns in the developing incisors, with robust expression in ameloblast and odontoblast layers. Strikingly, epithelium-specific *Cdc42* deletion resulted in enamel defects in incisors. Ameloblast differentiation was inhibited, and hypomineralization of enamel was observed upon epithelial *Cdc42* deletion. Proteomic analysis showed that abnormal mitochondrial components, phosphotransferase activity, and ion channel regulator activity occurred in the *Cdc42* mutant dental epithelium. Reactive oxygen species accumulation was detected in the mutant mice, suggesting that abnormal oxidative stress occurred after *Cdc42* depletion. Moreover, *Cdc42* mutant mice showed delayed tooth repair and generated less calcified enamel. Mitochondrial dysfunction and abnormal oxygen consumption were evidenced by reduced Apool and Timm8a1 expression, increased Atp5j2 levels, and reactive oxygen species overproduction in the mutant repair epithelium. Epithelium-specific *Cdc42* deletion attenuated ERK1/2 signaling in the labial cervical loop. Aberrant Sox2 expression in the mutant labial cervical loop after clipping might lead to delayed tooth repair. These findings suggested that mitochondrial dysfunction, up-regulated oxidative stress, and abnormal ion channel activity may be among multiple factors responsible for the observed enamel defects in *Cdc42* mutant incisors. Overall, Cdc42 exerts multidimensional and pivotal roles in enamel development and is particularly required for ameloblast differentiation and enamel matrix formation.

## Introduction

Dental enamel is the most calcified tissue in the human body.^1^ It bears heavy forces from mastication, wears from food, and protects dentin against erosion. The formation of enamel accompanies tooth development, which is initiated by invagination of the dental epithelium and condensation of the underlying mesenchyme. Enamel is secreted by ameloblasts of the tooth germ. After tooth eruption, the ameloblasts disappear; thus, the enamel hardly regenerates due to its ectodermal origination.^2^

Developmental defects of enamel (DDE), including hypoplasia and hypomineralization, affect a significant proportion of the population, with the possibility of defects appearing in both primary and permanent dentition.^3^ Among these defects, the prevalence of amelogenesis imperfecta ranges from 1/700 to 1/14,000. The average worldwide prevalence of molar-incisor hypomineralization is approximately 14%, with a large range of close to 0 to over 40%.^3^, ^4^, ^5^ Loss of enamel exposes the dentin in the oral cavity, which affects aesthetics. Moreover, teeth with enamel defects tend to suffer sensitivity and dental decay and may further require root canal treatment or extraction.

The prevalence of DDE differs in different populations. Clinical data demonstrate that hypoxia and malnutrition enhance the prevalence of DDE. It is 49.6%–50.6% in infants from a socially and economically poor population and that of prematurely born children is up to 75% in Brazil.^6^, ^7^, ^8^ Children with systemic diseases appear to have a higher prevalence of enamel defects.^9^ For example, children with congenital heart disease were observed to be more prone to enamel defects and periodontal diseases owing to a history of hypoxia in primary and permanent dentition.^10^ Enamel defects occur due to impaired ameloblast differentiation and enamel matrix deposition. Genetic changes such as KLK4 gene mutation in the dental epithelium lead to molar hypermineralization.^11^ In addition, during amelogenesis, environmental factors, including fluoride, can cause enamel hypoplasia by increasing reactive oxygen species (ROS) production in ameloblasts.^12^ Uncovering the interaction of genetic and environmental causes attributed to DDE helps with the prevention and prognosis of DDE.

The Rho GTPase family plays increasingly recognized roles in tooth development.^13^ Rho family GTPases, including RhoA, Rac1, and Cdc42, are expressed in the developing tooth organ and mediate the differentiation of ameloblasts and odontoblasts.^14^ Recently, the Rho GTPase family was found to participate in the cellular response to environmental stimuli.^15^ Unlike RhoA and Rac1, Cdc42 was found to be involved in cellular homeostasis and response to environmental stimuli or respiratory sufficiency.^16^^,^^17^ Cdc42 has also been reported to modulate mitochondrial polarity and ROS production.^18^^,^^19^ Our previous study demonstrated that Cdc42 was expressed in enamel organs and the underlying mesenchyme during tooth development, and *Cdc42* deletion induced cystogenesis in enamel organs with disrupted ameloblast polarity.^20^ Because the cell components are complicated during embryonic tooth development, the functional roles of Cdc42 in postnatal ameloblast differentiation and enamel matrix deposition require more evidence. Whether Cdc42 modulates cellular responses to environmental stimuli, such as incisor wear during amelogenesis, remains unclear. In this study, we demonstrated the role of Cdc42 in enamel development and tooth repair via inducible epithelium-specific knockout *Cdc42* mice. We found that *Cdc42* deletion yielded enamel defects (including impaired ameloblast differentiation and enamel hypomineralization), which is reminiscent of DDE. Loss of *Cdc42* arrested incisor repair by interrupting mitochondrial functions and altered cellular oxygen consumption by ROS production in dental epithelial stem cells.

## Materials and methods

### Generation of inducible epithelium-specific knockout *Cdc42* mice

Animal research was approved by the Sun Yat-sen University Institutional Animal Care and Use Committee (IACUC2022-001734&2023–000974, SYSU). *K14-cre/ERT* (Stock No. 005107) and *Cdc42*^*loxp/loxp*^ (Stock No. 027576) mice were acquired from the Jackson Laboratory. *K14-cre/ERT;Cdc42*^*loxp/+*^ mice were established by crossing *K14-cre/ERT* mice with *Cdc42*^*loxp/loxp*^ mice. The compound heterozygous mice were healthy and survived. *K14-cre/ERT;Cdc42*^*loxp/loxp*^ (*Cdc42*^*cKOER*^) mutant mice were generated by crossing *K14-cre/ERT;Cdc42*^*loxp/+*^ mice with *Cdc42*^*loxp/loxp*^ mice. Mouse genotyping was performed by general polymerase chain reaction (PCR) with the *Cdc42* primer (F5′-AGACAAAACAACAAGGTCCAGA-3′; R5′-GCACCATCACCAACAACAAC-3′) per the genotyping protocol from the Jackson Laboratory. Tamoxifen (Sigma, USA) was administered intraperitoneally to induce the deletion of *Cdc42* in dental epithelium components, and the dose of tamoxifen was referred to in other publications.^21^

### Micro-CT

Micro-CT was applied to assess enamel development. Mandibular incisors were dissected and preserved in 75% ethanol at 4 °C. The micro-CT data were acquired by a Scanco Medical μCT 50 System (Scanco Medical AG, Bassersdorf, Switzerland) at the following parameters: voltage, 70 kV; X-ray intensity, 200 μA; voxel size, 6 μm. Raw data were transformed into DICOM format and analyzed on MIMICS 17.0 (Materialise, Belgium). For mineralized analysis of enamel, the data of postnatal day 14 (P14) incisors were converted to DICOM format and analyzed on MIMICS 17.0. The sagittal views along the major axis of the lower incisor were set as the base plane among the samples. We adopted the 7200 Hounsfield unit as a threshold to highlight the highly mineralized area, which was supposed to be enamel.

### Histological analysis and Masson's trichrome staining

Mandibles were dissected and fixed in 4% paraformaldehyde for 24 h, followed by decalcification in 0.05 M EDTA (ethylenediaminetetraacetic acid) for 4 weeks. After dehydration in gradient concentrations of ethanol, the samples were embedded in paraffin and sectioned at 4.5 μm. A hematoxylin and eosin staining kit (Servicebio, China) and Masson's trichrome staining kit (Solarbio, China) were used according to the manufacturer's protocol.

### Proteomic analysis

Tamoxifen or corn oil was intraperitoneally injected to construct mutant or control *Cdc42*^*cKOER*^ mice. Molar tooth germs of P5 mice in both groups were dissected and subjected to LC‒MS/MS assays and LFQ quantification by Aksomics (Shanghai, China) as previously described.^22^ Briefly, we lysed the tooth germs in RIPA buffer with a protease inhibitor cocktail, and then ultrasonication was applied to extract the protein. After BCA quantification and sample preparation, LC‒MS/MS was run with the following parameters: MS1 resolution at M/Z 200: 70,000; MS2 resolution at M/Z 200: 17,500; MS1 automatic gain control at 3 × 10^6^; and MS2 automatic gain control at 1 × 10^5^. The measured data were processed using the UniProt-proteome-mouse-2021.2.fasta database on MaxQuant (2.0.1.0) software for protein identification. Proteins with expression fold change ratio A/B ≥ 2.0, *P* value ≤ 0.05, and unique peptide ≥2 or ratio A/B ≥ 2.0 and unique peptide ≥2 were defined as significantly differentially expressed and were then subjected to subsequent KEGG analysis.

### Immunofluorescence staining and confocal microscopy observation

Immunofluorescence staining was performed per previous studies.^20^ Briefly, mandibles were decalcified with 0.5 M EDTA as needed, with paraffin blocks sectioned at 5 μm thickness. Primary antibodies, including Amelogenin (1:50, Santa Cruz, USA), Apool (1:100, Affinity Biosciences, China), 4HNE (1:200, Bioss, China), Sox2 (1:200, Abcam, UK), Timm8a1(1:50, Proteintech, USA), and Cdc42 (1:100, Abcam, UK), were applied. Alexa Fluor secondary antibodies (A-21070, A-11005 Thermo Fisher, USA) were used to detect the signals. Sections were examined by confocal fluorescence microscopy (Olympus FV3000, Japan).

### Western blotting

Molar tooth germs of P5 mice or labial cervical loop (LaCL) of clipped and intact incisors were dissected under microscopy and then lysed in RIPA buffer with protease and phosphatase inhibitor cocktail to extract protein by ultrasonication. The protein concentration was measured by BCA assay. Western blotting was performed following the protocol recommended by the manufacturers. Primary antibodies were used as follows: Apool (1:1000, Affinity, China), Ndufs6 (1:1000, Affinity, China), Amelogenin (1:1000, Santa Cruz, USA), ERK1/2 (1:1000, Cell Signal Technology, USA), p-ERK1/2 (1:1000, Cell Signal Technology, USA), NP-4ebp1 (1:1000, Cell Signal Technology, USA), β-actin (1:1000, Cell Signal Technology, USA). The relative expression of target proteins was assessed by ImageJ (1.53c, NIH, USA).

### qRT‒PCR

Dental epithelium was isolated from tooth germs of different groups: group 1: P5 *Cdc42*^*cKOER*^ mice after tamoxifen injection; group 2: P5 *Cdc42*^*cKOER*^ mice after coin oil injection; group 3: mandibular lower incisors after clipping for 3 days with tamoxifen injection; group 4: mandibular lower incisors after clipping for 3 days with coin oil injection served as the controls. Total RNA was extracted using an RNA-Quick Purification Kit (Yishan Biotechnology, China) per the manufacturer's protocol. We synthesized cDNA with the PrimeScript RTreagen Kit (Takara, Japan). qRT‒PCR was performed on ABI QuantStudio5 using Hieff qPCR SYBR® Green Master Mix (Yeasen, China). The primers were as follows: *Timm8a1*, 5′-TATGGGACTGCTTTCTCAAGGTC-3′ and 5′-GTTGGATTGCAGGAACACTCC-3'; *Atp5j2*, 5′-CCGAGCTGGATAATGATGCG-3′ and 5′-TAATCCCCGAGATGCTGCCT-3'.

### ROS detection

The mutant and the control dental epithelium were harvested from P5 tooth organs or day 3 LaCL after incisor clipping. ROS levels were measured by a DCFH-DA probe as previously described.^23^ Briefly, tissues were dissected and treated with collagenase at 37 °C for 1.5 h. The tissues were then vortexed gently to homogenize the tissue in DMEM. The homogenate was then incubated in 50 μM DCFH-DA for 30 min in the dark. After incubation, the samples were centrifuged at 3000 rpm for 5 min to collect the cells, which were then resuspended in phosphate-buffered saline buffer twice. DCFH-DA fluorescence was detected at 488-nm excitation light and 525-nm emission light under a Biotek-Synergy H1 (Agilent, USA).

### Construction and measurement of a mouse incisor injury and repair model

Animal research was approved by the Sun Yat-sen University Institutional Animal Care and Use Committee (IACUC2022-001734&2023–000974, SYSU). A mouse incisor injury model was constructed as previously described.^24^ Briefly, after tamoxifen administration (coin oil administration served as the control) for 5 days in 6-week-old *Cdc42*^*cKOER*^ mice, the mandibular incisors were clipped at the gingival level. The renewal portion of clipping incisors was defined from the gingival margin to the tip of the incisor as described in a previous study.^24^ These mice were then followed for 7 days to observe regeneration of injured incisors. On day 3 and day 7 after incisor clipping, we utilized calipers to measure the length of regenerated incisors. The measurement was repeated 3 times for each mouse, and there were at least 3 mice in each group.

### EdU assay and TUNEL staining

At P5, *Cdc42*^*cKOER*^ mice were prepared after a 2-day injection of tamoxifen or corn oil for EdU and TUNEL staining. For the EdU assay, mice were intraperitoneally injected with 5 mg/kg EdU and sacrificed after 24 h according to the manufacturer's protocol (C00053, RiboBio, China). Mandibles were collected and treated with 4% paraformaldehyde and EDTA. After subsequent dehydration, the samples were then embedded and underwent frozen slicing at 8 μm thickness. Signals were detected by a Cell-Light EdU Apollo567 In Vitro Kit (C10301-1, RiboBio, China). For TUNEL staining, a TUNEL staining kit (C1089, Beyotime, China) was used per the manufacturer's protocol. The fluorescence signals were examined under a confocal microscope (Olympus FV3000, Japan).

### Statistical analysis

All experiments were performed in biological triplicates. All quantitative data upon confirmation of normal data distribution were expressed as mean ± standard deviation and analyzed by Student's *t*-test or analysis of variance with Bonferroni correction, with significance of *P* < 0.05.

## Results

### Cdc42 expression in postnatal tooth development

**Figure 1:**
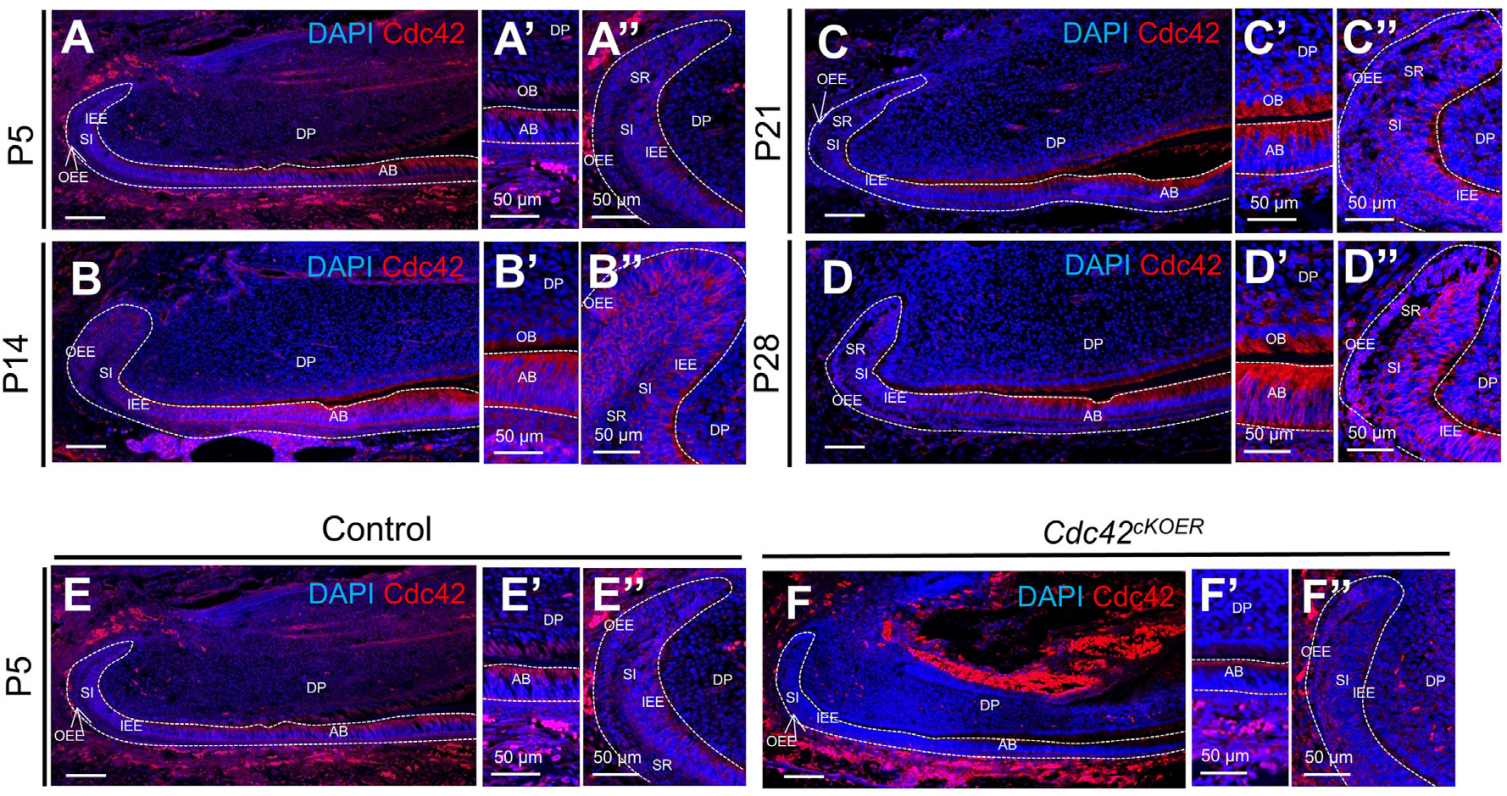
Cdc42 expression in postnatal incisors and generation of *Cdc42*^*cKOER*^ mice. **(A–E**) Immunofluorescence staining of Cdc42 (red) in the mandibular incisors of wild-type mice from postnatal day 5 to day 28. **(A’–E’)** Magnification of ameloblasts in panels A–E. **(A’’–E’’**) Magnification of LaCL in panels A–E. **(E,F,’,’’**) Immunofluorescence staining of Cdc42 in *Cdc42*^*cKOER*^ mice after tamoxifen injection. Scale bars: 100 μm. AB, ameloblasts; DP, dental papilla; IEE, inner enamel epithelium; OB, odontoblasts; OEE, outer enamel epithelium; SI, stratum intermedium; SR, stellate reticulum; LaCL, labial cervical loop.

Our previous study revealed the expression pattern of Cdc42 in multiple dental epithelial compartments and dental papilla from embryonic day 11 to P0.^20^ We further mapped the level of Cdc42 at postnatal day 5 (P5), P14, P21, and P28 by immunofluorescence (Fig. 1). Cdc42 continued to be expressed in the dental epithelium and dental papilla after birth in incisors. At P5, Cdc42 was highly expressed in mature ameloblasts (Fig. 1A, A’) and relatively lower in the cervical loop than in mature ameloblasts (Fig. 1A, A’’). Moreover, Cdc42 was located in all epithelial compartments, including the inner enamel epithelium, outer enamel epithelium, stellate reticulum, and stratum intermedium layers, in the cervical loop, showing a consistent pattern among P5, P14, P21, and P28 incisors (Fig. 1A”, B’’, C’’, D’’). Cdc42 expression increased with cell polarization in both the dental epithelium and dental mesenchyme, indicating robust expression of Cdc42 in ameloblasts and odontoblasts (Fig. 1A’, B’, C’, D’). Although K14 is located in different epithelial tissues, it is considered an odontogenic marker in most cells of enamel organs, especially in ameloblasts, during embryogenesis.^20^^,^^25^ Consequently, we utilized inducible K14-cre to construct an epithelium-specific *Cdc42* knockout mouse model aiming to target Cdc42's functional roles in ameloblast differentiation and enamel formation. After tamoxifen injection for 2 days in P5 *K14-cre/ERT;Cdc42*^*loxp/loxp*^ mice, lower fluorescence was observed in dental epithelial compartments in the mutant mice than in the littermate controls (Fig. 1E, F), confirming the successful deletion of *Cdc42* specifically in the dental epithelium.

### Enamel defects upon epithelial depletion of *Cdc42* during tooth development

**Figure 2:**
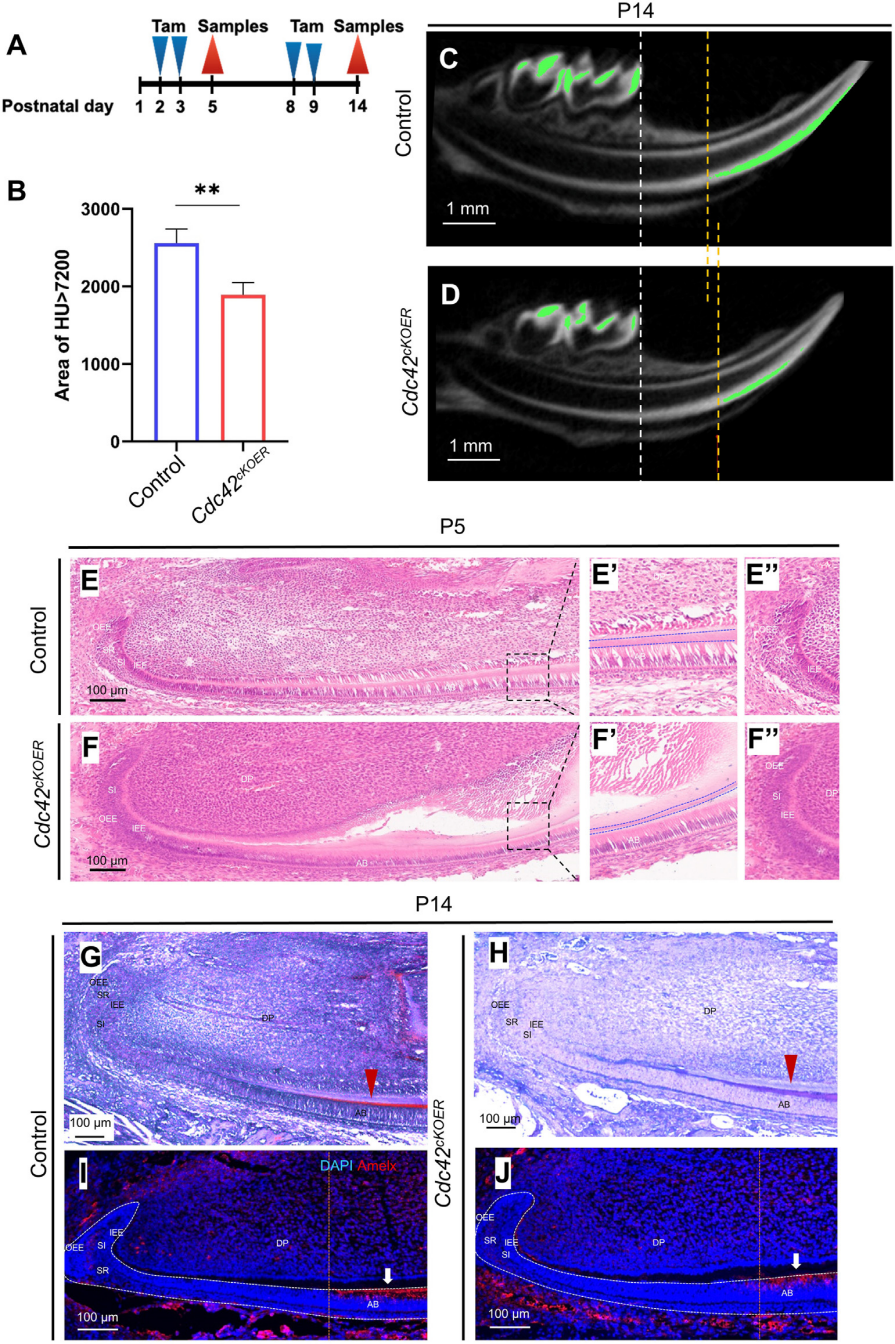
Depletion of *Cdc42* yielded hypomineralization enamel and impaired ameloblast differentiation. **(A)** Schematic illustration of the tamoxifen administration plan and the strategy to establish epithelium-specific *Cdc42* deletion during postnatal tooth development. **(B)** Quantification of the enamel area of Hounsfield unit >7200 from micro-CT data in the control and *Cdc42*^*cKOER*^ mice at postnatal day 14. The values were presented as mean ± standard deviation. ^∗∗^*P< 0.01*. **(C, D)** Micro-CT images of mandibles at postnatal day 14 in the control and *Cdc42*^*cKOER*^ mice. Green signals: Hounsfield unit >7200. **(E, F)** Hematoxylin and eosin staining of lower incisors at postnatal day 5 in the control and mutant mice. **(E’,F’)** Magnification of ameloblasts, enamel, and dentin in panels E and F. **(E’’,F’’)** Magnification of LaCL in panels E and F. **(G, H)** Masson's trichrome staining of the lower incisors at postnatal day 14 in the control and mutant mice. **(I, J)** Immunofluorescence staining for Amelx in the control and *Cdc42*^*cKOER*^ lower incisors at postnatal day 14. AB, ameloblasts; DP, dental papilla; IEE, inner enamel epithelium; OEE, outer enamel epithelium; SI, stratum intermedium; SR, stellate reticulum; LaCL, labial cervical loop.

To further investigate the role of Cdc42 in enamel formation after birth, we induced the deletion of *Cdc42* via tamoxifen administration at P2–P3 and P8–P9 in *K14-cre/ERT;Cdc42*^*loxp/loxp*^ mice (*Cdc42*^*cKOER*^) (Fig. 2A). At P14, the mandibles were harvested to perform micro-CT scanning. The molars and incisors erupted, and there were no significant differences in tooth morphology in either mutant or control mice (Fig. 2C, D). Then, we set up a 7200 Hounsfield unit as a threshold to highlight the highly mineralized area in P14 mandibles. The results showed lower intense signals (green signals) in the enamel of the mutant molars and incisors compared with those in the control, which were reminiscent of enamel hypomineralization (Fig. 2B–D). Less enamel matrix was secreted by *Cdc42*^*cKOER*^ ameloblasts at P5 compared with that in the control (Fig. 2E, E’, F, F’). The borders of the stratum intermedium layer, stellate reticulum layer, and inner enamel epithelium layer were blurred in the mutant LaCL by hematoxylin and eosin staining examination, which was similar to our previous study.^20^ Masson's trichrome staining showed that less enamel stained red at P14 mutant incisors, while red staining of the enamel matrix was easy to recognize in the control incisors (Fig. 2G, H). Moreover, fewer amelogenin-positive cells in the dental epithelium after loss of *Cdc42* (Fig. 2I, J) indicated impaired ameloblast differentiation in the mutant mice, similar to enamel hypoplasia.

### Altered mitochondrial respiratory chain in the *Cdc42* mutant epithelium

**Figure 3:**
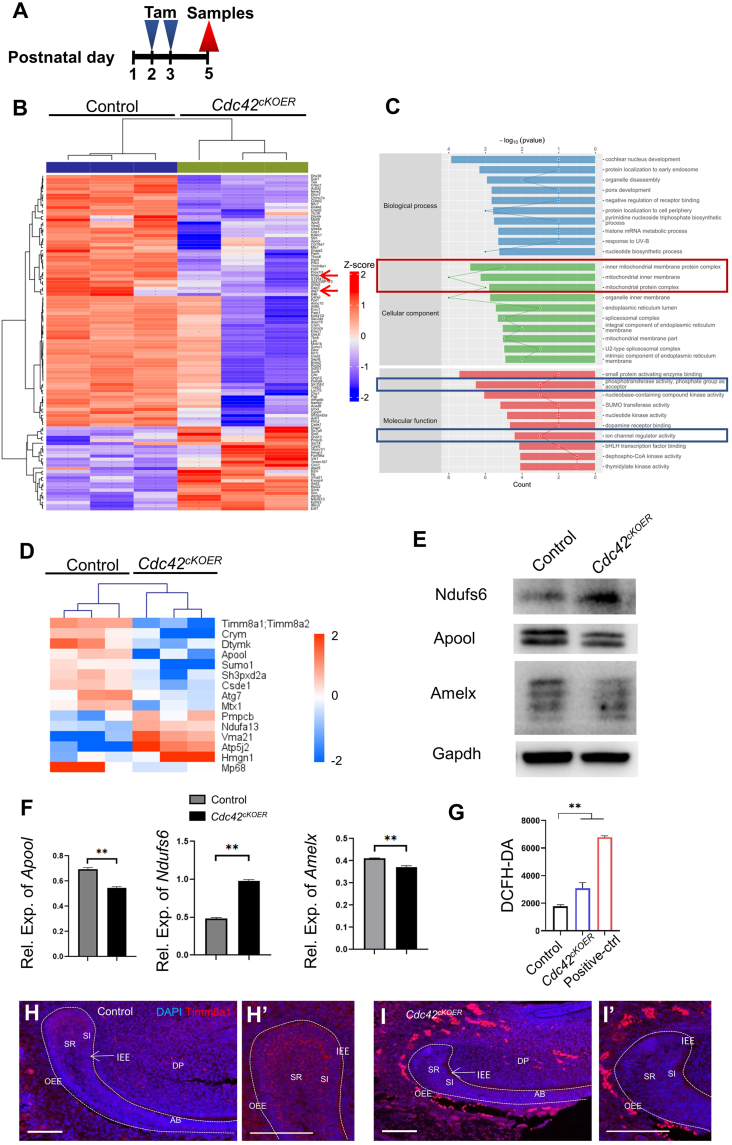
Proteomic and reactive oxygen species valuation of *Cdc42*^*cKOER*^ dental epithelium. **(A)** The workflow of tamoxifen administration and sample collection. **(B)** Altered expression of protein from 3 samples in the control and mutant groups. Red arrows: Rheb and Atg7. **(C)** KEGG analysis of the differential expression of proteins. Red square: protein relative to mitochondria. Blue squares: protein relative to phosphotransferase activity and ion channel regulator activity. **(D)** Specific proteins modulating mitochondrial functions in the control and *Cdc42*^*cKOER*^ dental epithelium. **(E, F)** Western blot and qRT‒PCR measurement of Ndufs6, Amelx, and Apool expression levels. **(G)** Reactive oxygen species levels determined by DCFH-DA. **(H–I’)** Immunofluorescence staining of Timm8a1 in the control and mutant incisors at postnatal day 5. Scale bars, 100 μm. The values were presented as mean ± standard deviation. ^∗∗^*P< 0.01*.

To elucidate the mechanism of enamel defects upon depletion of *Cdc42*, we isolated dental epithelium from the P5 mutant mice after tamoxifen injection and the control mice and then performed proteomic analysis (Fig. 3A). Gene ontology analysis showed that mitochondrial components were changed in the *Cdc42*^*cKOER*^ dental epithelium compared with the controls (Fig. 3C). Mitochondria, as the major site of oxidative reactions, provide the majority of the energy needed by aerobic cells to maintain their physiological activity.^26^ Specifically, groups of proteins belonging to the mitochondrial protein complex and inner mitochondrial protein complex, such as Timm8a1/Timm8a2, Apool, and Ndufa13, exhibited differential expression in the mutant dental epithelium, indicating an abnormal mitochondrial structure in the mutant dental epithelial cells compared with the controls (Fig. 3D). Lower levels of Apool and higher levels of Ndufs6 were measured by Western blot and qRT‒PCR (Fig. 3E, F), demonstrating that the mitochondrial respiratory chain was changed in *Cdc42*^*cKOER*^ mice. Our further results of ROS levels in the dental epithelium revealed the overproduction of ROS in *Cdc42*^*cKOER*^ mice, which is consistent with mitochondrial dysfunction, suggesting the pivotal roles of Cdc42 in epithelial cell respiration (Fig. 3G). Immunofluorescence staining of Timm8a1 in the dental epithelium showed lower signals in the mutant mice than in the controls (Fig. 3H–I’), consistent with Figure 3D.

We also recognized abnormal expression of proteins relative to phosphotransferase activity and ion channel regulator activity after *Cdc42* deletion. These molecular functions are involved in protein synthesis and enamel mineralization (Fig. 3C). Rheb and Atg7 decreased in the *Cdc42*^*cKOER*^ epithelium, revealing that there might be deviant autophagy-related metabolism in the cells (Fig. 3B).

### Restraint incisor repair via loss of *Cdc42* in the dental epithelium

Mouse incisors erupt between P10 and P12. Once erupted, the upper and lower incisors undergo chewing, and the enamel begins to wear. Mouse incisors wear down and completely renew their four incisors every 35–45 days. Studies have shown that mouse incisors are especially useful for studying postnatal enamel development and the mechanism by which adult stem cells regenerate tissues.^13^ During tissue repair, stem cells seem to be more active and accelerate cellular metabolism, which requires more energy from mitochondria.^27^

**Figure 4:**
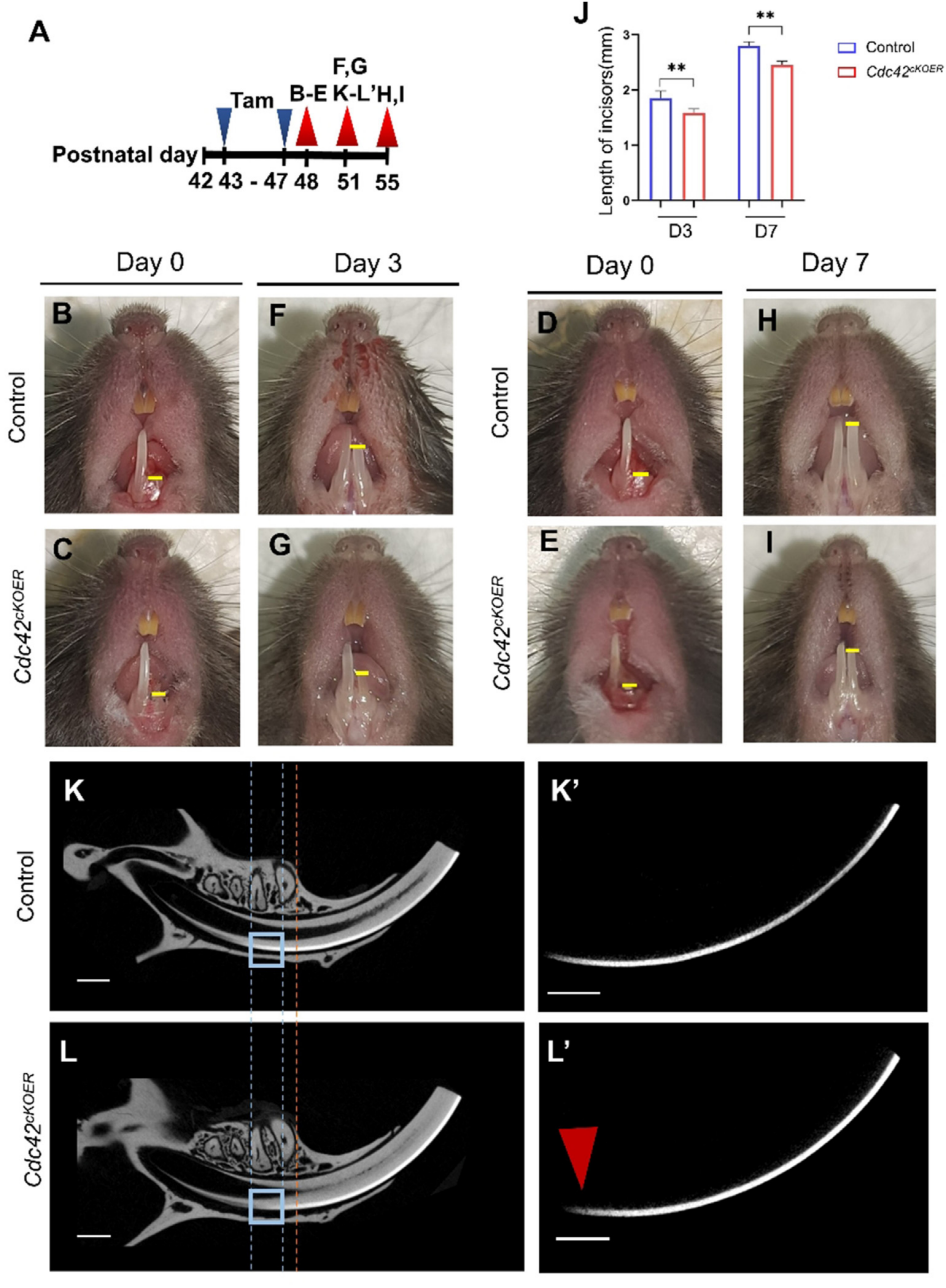
Construction and measurement of a mouse incisor regeneration model with or without *Cdc42* deletion. **(A)** Schematic diagram of tamoxifen injection and sample collection in the establishment of incisor clipping models. **(B–I)** Images of incisor clipping in wild-type and *Cdc42*^*cKOER*^ mice. **(K,K’,L,L ’)** Micro-CT results of mandibles and enamel in the control and *Cdc42*^*cKOER*^ mice after 3 days of clipping. Orange dashed line: the most mesial point of the lower first molar set as the plane of reference. Blue dashed lines and blue squares: the area showing the difference between the control and the mutant groups. Red arrowhead: less and hypomineralized enamel matrix upon epithelium-specific *Cdc42* depletion. **(J)** Quantification of the regenerated incisor length in panels B–I. Scale bars,100 μm. The value was presented as mean ± standard deviation. ∗∗*P< 0.01*.

Aiming to uncover whether a lack of *Cdc42* impaired cellular respiration via oxidative metabolism in the dental epithelium during incisor regeneration after masticatory wear, we constructed an incisor injury repair mouse model using *K14-cre/ERT;Cdc42*^*loxp/loxp*^ mice according to Yu's article to mimic tooth repair.^24^ Tamoxifen administration and incisor-clipping-sample collection are shown in Figure 4A. The photographs and statistical analysis showed that *Cdc42* deletion caused robust impaired incisor regeneration 3 days after incisor clipping (Fig. 4F, G, J). Until day 7 after clipping, *Cdc42*^*cKOER*^ mice could regenerate most of the length of the lower incisor. However, the length in mutant mice was shorter than that of the controls (Fig. 4H–J). Micro-CT was used to analyze the length and intensity of enamel. The results demonstrated less enamel formation and lower calcification intensity in the mutant incisor after clipping for 3 days (Fig. 4K–L’), indicating that depletion of *Cdc42* in the dental epithelium delayed mouse incisor repair and formed less and hypomineralized enamel.

### Disruption of *Cdc42* arrested dental epithelial stem cell differentiation during tooth repair

**Fig. 5:**
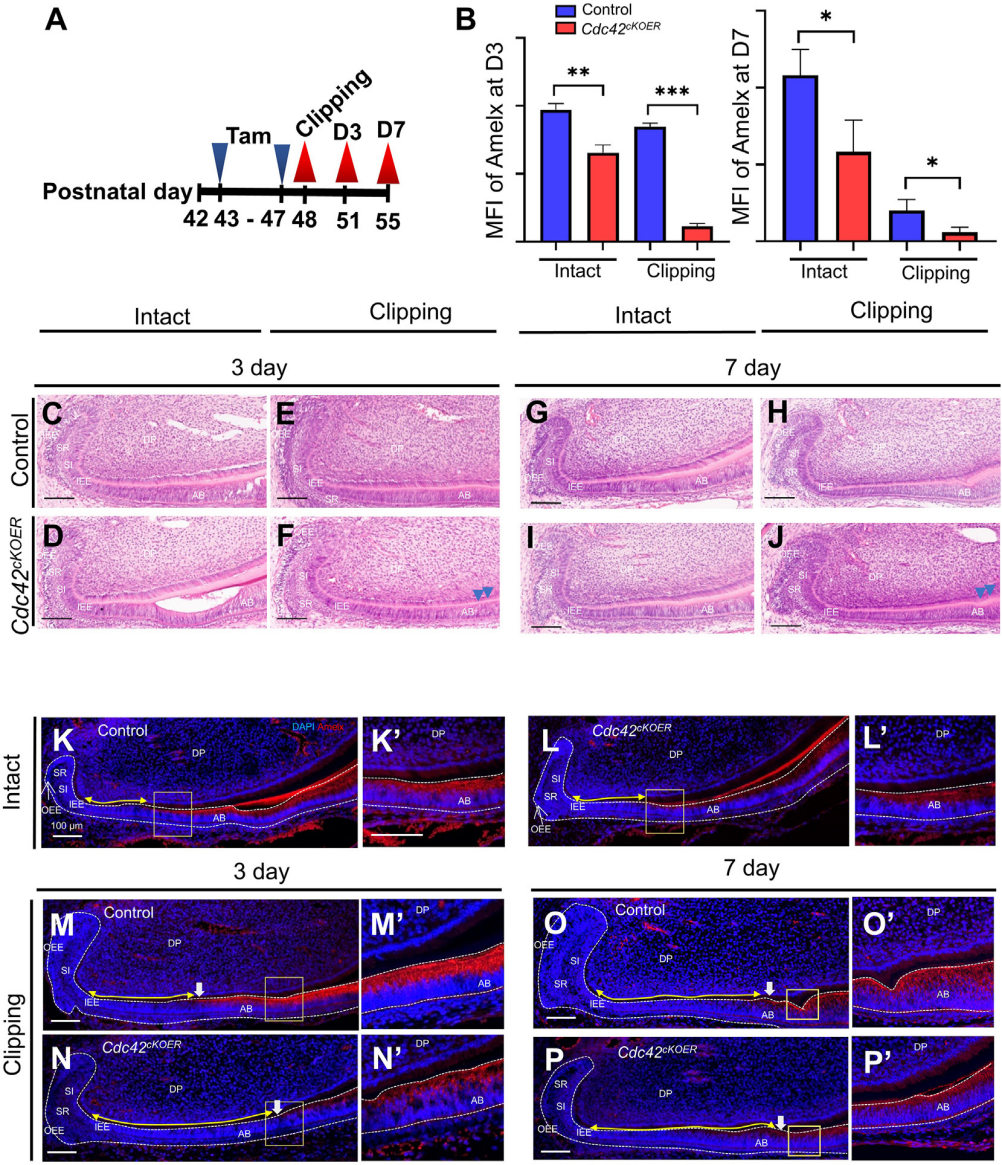
Histology analysis of incisor repair in control and *Cdc42*^*cKOER*^ mice. **(A)** The workflow of tamoxifen injection, incisor clipping, and sample collection. **(B)** Quantification of the mean fluorescence intensity of the Amelx in the incisors after 3 days or 7 days of clipping. The values were presented as mean ± standard deviation. ^∗∗∗^*P* < 0.001. **(C**–**J)** Hematoxylin and eosin staining of the labial cervical loop in the control and *Cdc42*^*cKOER*^ mice with/without incisor clipping for 3 days and 7 days. **(K, L)** Immunofluorescence staining of Amelx in the control and mutant incisors without clipping. **(K’,’)** Magnification of the yellow squares in panels K and L. **(M**–**P)** Immunofluorescence staining of Amelx in the control and mutant incisors after clipping for 3 days and 7 days. **(M’ –P’)** Magnification of the yellow squares in M–P. Scale bars: 100 μm. AB, ameloblasts; DP, dental papilla; IEE, inner enamel epithelium; OEE, outer enamel epithelium; SI, stratum intermedium; SR, stellate reticulum.

To address how Cdc42 regulated incisor repair, we further performed histological analysis on day 3 and day 7 after incisor clipping in both control and mutant mice (Fig. 5). After 5 days of tamoxifen administration, the mutant LaCL presented less organized inner enamel epithelium, stratum intermedium, stellate reticulum, and ameloblast layers than the controls (Fig. 5C, D, G, I), consistent with P5 mutant incisors (Fig. 2). After incisor clipping for 3 days, hematoxylin and eosin staining demonstrated that LaCL structures in the control group were similar to those in the no-clipping group (Fig. 5C, E). However, the mutant ameloblasts deposited less enamel matrix (Fig. 5F, J, arrowheads) compared with that of the control (Fig. 5E, H). Amelogenin (Amelx) is the most abundant protein in secretory-stage enamel.^28^ Immunofluorescence staining showed that in the control mice, after the establishment of clipping, preameloblasts, which were Amelx-negative, were elongated on day 3 (Fig. 5K, M) and day 7 (Fig. 5O). The number of preameloblasts increased after clipping, which suggested that the epithelial stem cells had proliferated and tended to differentiate into ameloblasts aiming to repair the clipping incisor. Despite fewer Amelx-positive ameloblasts in the mutant incisor without clipping, cutting the incisor still elongated the pAB zone in the dental epithelium (Fig. 5N, P). The pAB zone in the mutant mice seemed to stretch closer toward the occlusal plane than that in the controls (Fig. 5M−P). The longer pAB zone and lower Amelx expression in the mutant incisors indicated arrested mature ameloblast differentiation after *Cdc42* deletion.

### Dysregulated oxidative stress via mitochondrial dysfunction upon epithelium-specific *Cdc42* deletion

**Figure 6:**
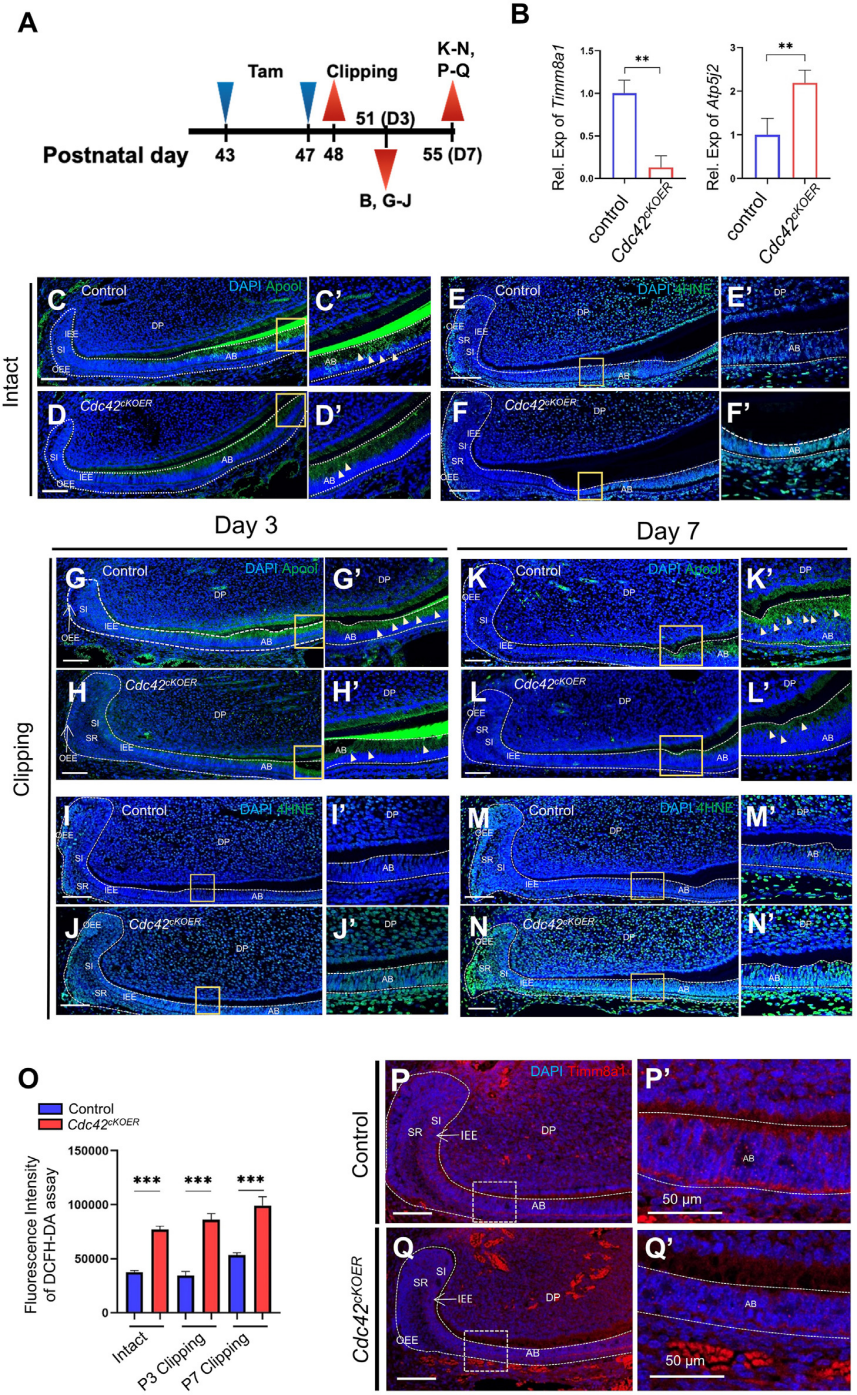
Analysis of mitochondrial functions and reactive oxygen species level determination in the incisor repair model. **(A)** Schematic figure of the incisor repair model and the time point at which mice were sacrificed. **(B)***Timm8a1* and *Atp5j2* expression measured by qRT‒PCR. The values were presented as mean ± standard deviation. ^∗∗^*P* < 0.01. **(C, D, G–J)** Immunofluorescence staining for Apool without incisor clipping (C, D) and with incisor clipping for 3 days (G, H) and 7 days (I, J). **(E, F, K–N)** Detection of 4HNE by immunofluorescence staining without incisor clipping (E, F) and with incisor clipping for 3 days (K, L) and 7 days (M, N). **(C’–N’)** Magnified images of yellow-boxed areas in panels C–N. **(O)** Reactive oxygen species evaluation in the dental epithelium after 3 days or 7 days or without clipping by DCFH-DA. **(P–Q′)** Detection of Timm8a1 by immunofluorescence staining with incisor clipping for 7 days in control and mutant mice. Scale bars in C–N’, P, Q: 100 μm, ^∗∗∗^*P* < 0.001. AB, ameloblasts; DP, dental papilla; IEE, inner enamel epithelium; OEE, outer enamel epithelium; SI, stratum intermedium; SR, stellate reticulum.

Next, we isolated dental epithelium 3 days after incisor clipping from *Cdc42*^*cKOER*^ mutant incisors and control incisors (Fig. 6A). The qRT‒PCR results demonstrated higher levels of *Atp5j2* and lower levels of *Tim8a1* in the mutant mice than in the control epithelium (Fig. 6B). In the littermate control mice, less Apool expression in ameloblasts and lower 4HNE (an oxidative/nitrosative stress biomarker) levels after clipping for 3 days suggested the adaptation of oxygen consumption through mitochondria during incisor repair (Fig. 6C, G, E, I). On day 7 after clipping, the wild-type incisor almost sustained Apool and 4HNE levels in the clipping groups (Fig. 6K, M) because incisor regeneration was not finished until day 14 according to references. After incisor impairment for 3 days and 7 days, less Apool was located in the mutant dental epithelium without clipping compared with that in the clipping controls, suggesting mitochondrial dysfunction during tissue repair (Fig. 6H, L). Increasing 4HNE congregated in the mutant epithelium, especially in the preameloblasts and ameloblasts, revealing higher oxidative stress due to mitochondrial dysfunction after *Cdc42* deletion (Fig. 6J, N). Levels of ROS were also measured by DCFH-DA assay. The results showed that more ROS were maintained in the mutant epithelium during incisor repair (Fig. 6O). Moreover, the mitochondrial inner membrane protein Timm8a1 sustained lower levels in the mutant LaCL and ameloblasts relative to those in the controls (Fig. 6P–Q’).

### Fewer Sox2^+^ stem cells and loss of *Cdc42* targeted ERK1/2 signaling in LaCL during incisor repair

**Figure 7:**
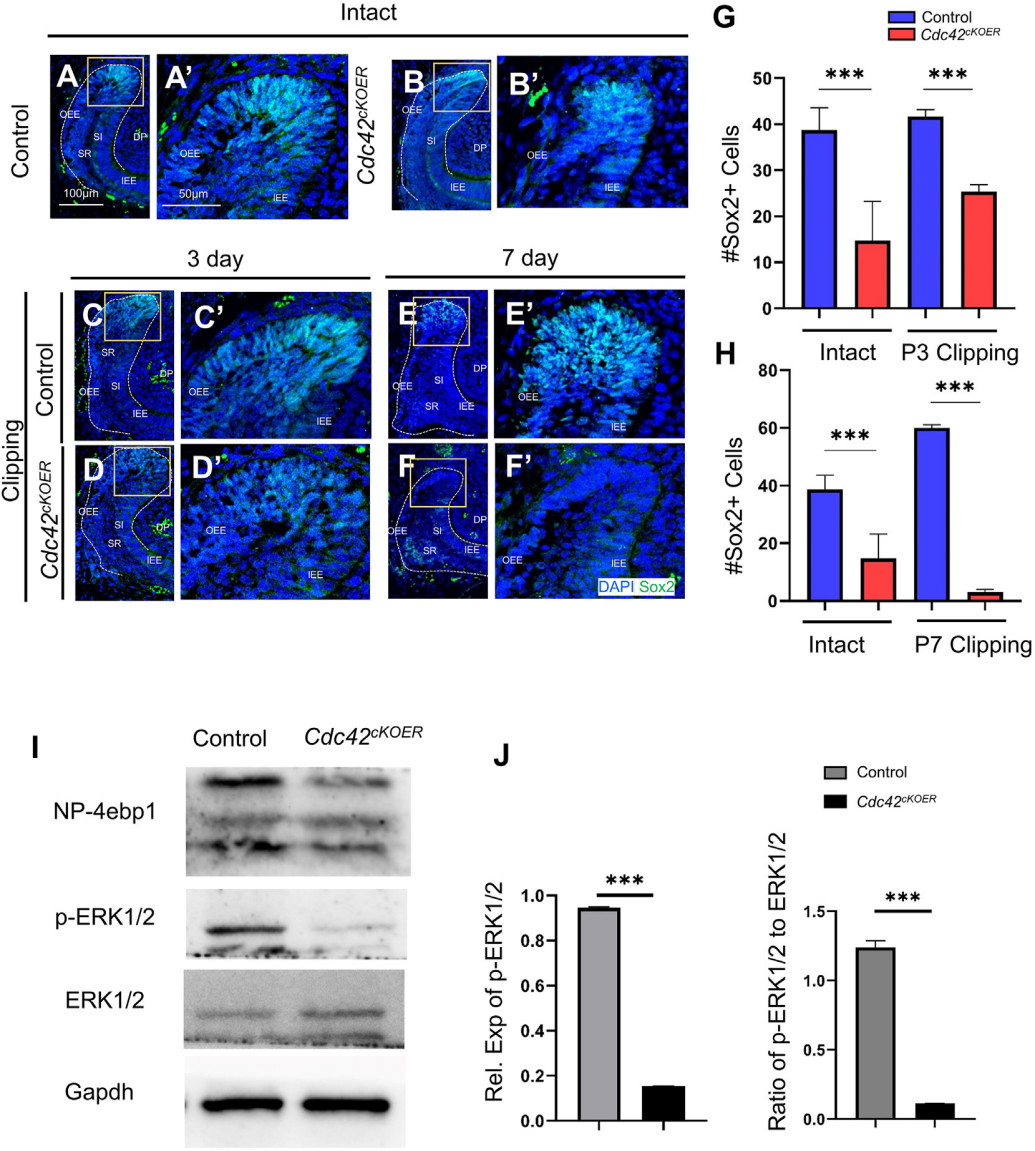
Mechanism of enamel defects induced by specific *Cdc42* knockout. **(A**–**F)** Immunofluorescence of Sox2 in labial cervical loops without clipping or with clipping for 3 days and 7 days. The labial cervical loops are illustrated by a dotted line. **(A’–H’)** Magnified views of the yellow boxed areas in panels A–F. **(G, H)** Quantification of Sox2-positive cells. The values were presented as mean ± standard deviation. ^∗∗∗^*P* < 0.001. **(I)** Expression of NP-4ebp1, p-ERK1/2, ERK1/2, and β-actin by Western blot in the control and *Cdc42*^*cKOER*^ mice after incisor repair for 3 days. **(J)** Quantification of p-ERK1/2 and p-ERK1/2/ERK1/2 from Western blot results by ImageJ. The values were presented as mean ± standard deviation. ^∗∗∗^*P* < 0.001. Scale bars: 100 μm. AB, ameloblasts; DP, dental papilla; IEE, inner enamel epithelium; OEE, outer enamel epithelium; SI, stratum intermedium; SR, stellate reticulum.

Sox2-positive cells were considered to contribute to all epithelial lineages of the tooth as progenitor cells.^29^ Aiming to study the mechanism of delayed tooth repair and enamel defects upon epithelium-specific *Cdc42* deletion, we marked Sox2-positive cells in incisors by immunofluorescence staining. The results showed that Sox2 remained positive in the LaCL stem cell niches in the wild-type mice before and 3 days after clipping (Fig. 7A, A’, C, C’). The number of Sox2^+^ cells in the mutant LaCL was less than that in the control LaCL (Fig. 7B, B’). After tooth clipping for 3 days, lower fluorescence of Sox2 was observed in the mutant incisor than in the control incisor (Fig. 7D, D’). Sox2 was hardly detected in the mutant LaCL on day 7 after clipping, while it remained positive in the control group (Fig. 7F, F’). The number of Sox2^+^ stem cells is shown in Figure 7G and H.

**Figure S1:**
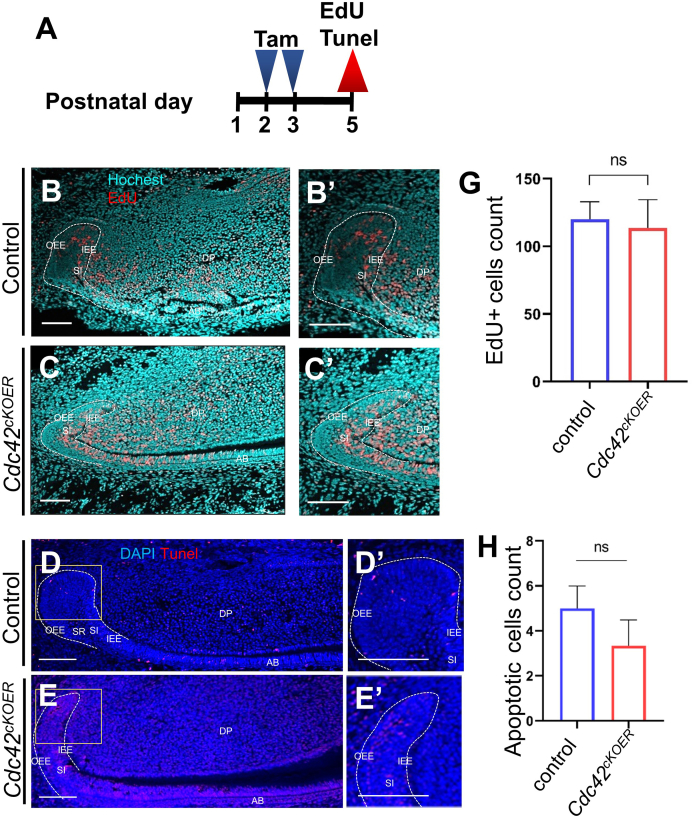
Cell proliferation and apoptosis in the control and mutant lower incisors at postnatal day 5. **(A)** Schematic diagram of tamoxifen administration and mouse sacrifice. **(B, C)** EdU staining for cell proliferation in the control and mutant incisors. **(B′, C′)** Magnification of the labial cervical loop in panels B and C. **(D, E)** TUNEL staining for apoptosis in the control and mutant incisors. **(D′, E′)** Magnification of the labial cervical loop in panels D and E. **(G, H)** Quantification of EdU^+^ and apoptotic cells in the control and mutant groups. The values were presented as mean ± standard deviation. Scale bars: 100 μm. ns: no significant difference. AB, ameloblasts; DP, dental papilla; IEE, inner enamel epithelium; OEE, outer enamel epithelium; SI, stratum intermedium; SR, stellate reticulum.

To verify the mechanism of Cdc42's role in mitochondria, we isolated dental epithelium after clipping for 3 days and performed western blotting. Nonphospho-4ebp1 (NP-4ebp1) and p-ERK1/2 were robustly decreased in the mutant mice relative to the controls (Fig. 7I, J). These results were consistent with our previous proteomic analysis in Figure 2 showing less expression of Rheb and Atg7 after deletion of *Cdc42*. We also performed EdU and TUNEL staining in the stem cell niche. However, there was no significant difference in cell proliferation and apoptosis between the mutant and control groups (Fig. S1).

## Discussion

How to treat enamel defects remains a clinical challenge for dentists. The study of enamel restoration can be categorized into three aspects. First, the microstructures and chemical composition of enamel are uncovered to construct biomimetic materials of enamel.^30^ Second, the signaling pathways of tooth development are well studied, aiming to utilize them for tooth regeneration or enamel generation.^31^^,^^32^ Last but not least, the mechanism of enamel formation, including amelogenesis and matrix deposition or mineralization, is considered to be a crucial component of research on enamel.^13^^,^^33^^,^^34^ Among all the strategies, the prevention of DDE during development is preferable. Our studies demonstrate an underlying network referring to Cdc42 and oxygen consumption via mitochondria during postnatal tooth development and repair, which would provide some insights into the mechanism of DDE.

Cdc42 plays a pivotal role in postnatal tooth development and tooth repair. Lack of *Cdc42* impairs postnatal ameloblast differentiation and reduces enamel matrix secretion, consistent with our previous study on prenatal tooth development.^20^ Less enamel matrix, together with lower calcification of enamel in the *Cdc42*^*cKOER*^ mice, are among the phenotypes of enamel defects after birth. The association of *Cdc42* mutations or abnormal activation with abnormal mineralization or other defects was revealed in multiple organ systems.^35^, ^36^, ^37^, ^38^ Whether enamel defects are indeed molar-incisor hypomineralization, enamel hypoplasia, or both requires further study. Our findings demonstrate that deletion of *Cdc42* yielded enamel defects and delayed tooth repair. Impaired ameloblast differentiation, decreased enamel matrix formation, and secreted enamel with lower calcification are the main causes of enamel defects in *Cdc42*^*cKOER*^ mice.

The pathogenesis of enamel defects may involve reduced cell proliferation, increased cell apoptosis, impaired ameloblast differentiation, and enamel malformation. *Cdc42* deletion leads to increased apoptosis in the primary enamel knot in enamel organs during tooth development on prenatal days, while depletion of *Cdc42* in the postnatal dental epithelium does not affect cell survival and proliferation.^20^ Therefore, enamel defects were mainly caused by the dysfunction of ameloblasts and hypomineralized enamel in the *Cdc42*^*cKOER*^ mice. Cell proliferation and differentiation require mitochondrial metabolism, particularly oxidative phosphorylation.^39^ Tooth development under hypoxia enhances the clinical prevalence of DDE.^40^ Less enamel matrix and lower calcified enamel demonstrated impaired ameloblast differentiation upon epithelium-specific *Cdc42* deletion. Ameloblast differentiation requires energy, and mitochondria are involved in Ca^2+^ signaling.^41^ The pronounced alteration of mitochondrial components (Apool, Timm8a1, *etc*.) and overproduction of ROS in the mutant epithelium indicated the disruption of cellular respiration, leading to abnormal energy metabolism in ameloblasts during amelogenesis. Our studies not only demonstrated the energy requirement from cellular aerobic metabolism during tooth repair but also revealed the functional role of Cdc42 in energy metabolism relative to cellular respiration during amelogenesis.

How Cdc42 regulates ameloblast mitochondria during cellular respiration might lurk in the functional role of Cdc42 in mitochondrial biogenesis and polarity. Loss of *Cdc42* yields lower phosphotransferase activity, which may decrease the energy needed for activation of ERK1/2 signaling. Fundamentally, inhibition of ERK reduces the transcription of genes related to mitochondrial biogenesis.^42^ ERK1/2, PKA, and CaMKII can phosphorylate dynamin-related protein 1, the controlling element of mitochondrial fission, at Ser616 and promote mitochondrial fission.^23^^,^^43^ The phosphorylation of dynamin-related protein 1 was reported to reduce pathological mitochondria and ROS generation and eventually improve cellular existence.^23^ Mitochondrial fission and fusion play a critical role in cellular metabolism dynamics. Previous research has indicated that the fission process might decrease the quality of mitochondria and therefore inhibit cellular respiration.^44^ In the context of high energetic demand or high oxidative stress, mitochondria may be significant in regulating the quality of aging or pathological mitochondria, as well as in reproducing robust mitochondria to maintain mitochondrial homeostasis and meet the consumptive energy requirements.^23^ On the other hand, activation of Cdc42 also acts as a binding partner of NDRG1, which regulates mitochondrial fission during fasting, and as the key regulator of mitochondrial polarity, which is crucial for cellular respiration.^18^^,^^45^ Thus, Cdc42 might regulate mitochondria directly or indirectly through ERK1/2 signaling in ameloblasts. Moreover, an efficient system of ion transport is a prerequisite for the biomineralization of enamel.^46^ Ion transportation, evidenced by the abnormal expression of proteins relative to ion channel regulator activity after *Cdc42* depletion, might be among the main causes of enamel hypomineralization.

Although the loss of *Cdc42* did not affect cell proliferation or apoptosis in postnatal incisors, it limited the renewal of Sox2-positive stem cells in the LaCL during tooth repair. Sox2^+^ cells are known to contribute to all epithelial lineages of the tooth.^29^ *Cdc42* deletion dislocates Sox2^+^ cells in the molar enamel organ during embryonic development.^20^ In neuron development, the proliferation of Sox2^+^ neural progenitor cells is modulated by Cdc42.^47^ The reduced number of Sox2^+^ stem cells in LaCL might be among the causes of shorter repair incisors in the mutant mice. However, the mechanism by which Cdc42 targets Sox2 requires further study.

**Fig. 8:**
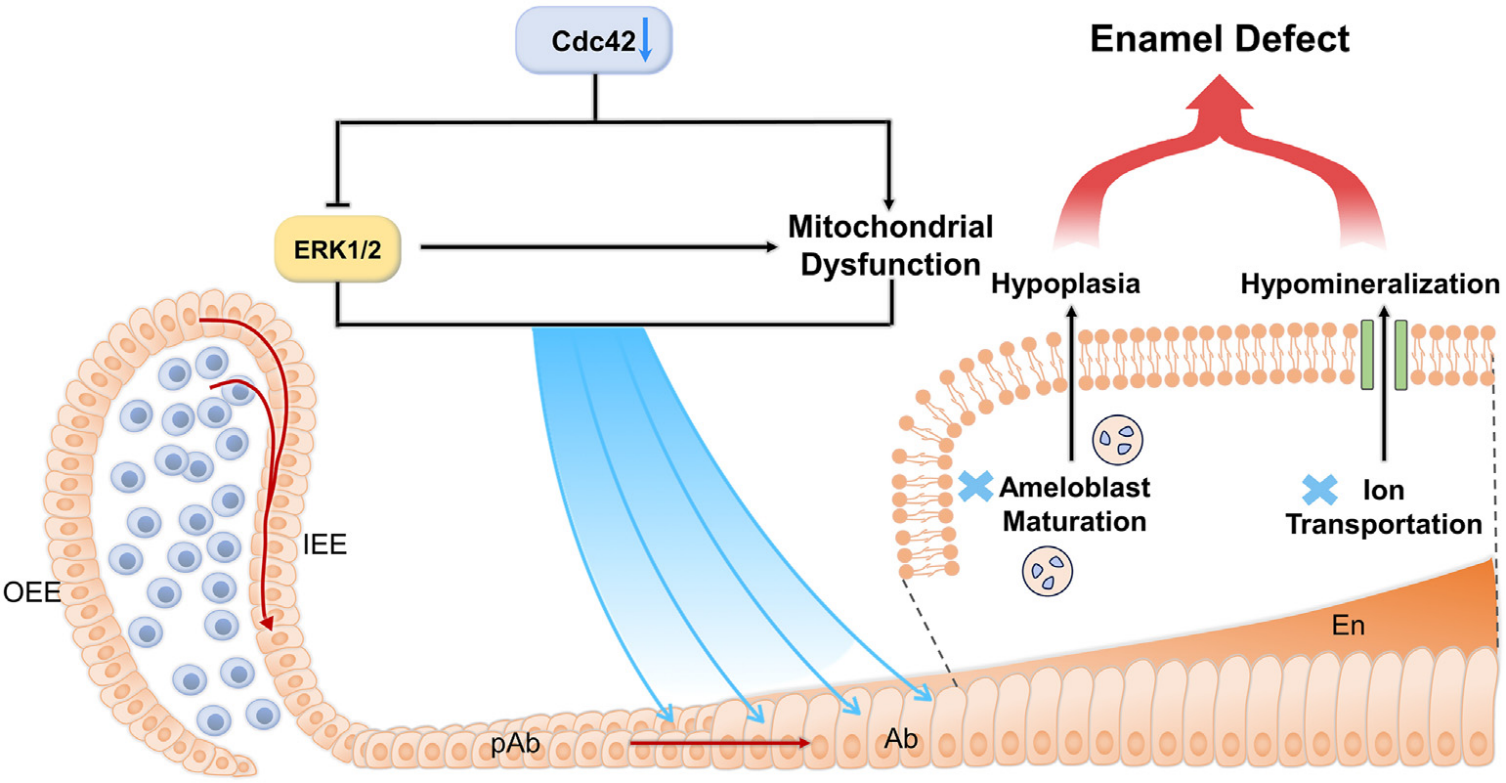
Schematic image of the mechanism of enamel defects upon loss of *Cdc42* in the dental epithelium after birth.

Taken together, the mechanism of enamel defects upon epithelium-specific *Cdc42* deletion could be demonstrated below (Fig. 8). *Cdc42* deletion attenuated ERK1/2 signaling, leading to impaired ameloblast differentiation. Moreover, the lack of *Cdc42* induced mitochondrial dysfunction directly or through the ERK1/2 signaling pathway. Last but not least, impaired ameloblast maturation and aberrant ion transportation might be among the causes of hypoplasia and hypomineralization, which are two aspects of enamel defects, as a consequence of *Cdc42* depletion. Consequently, Cdc42 exerts multidimensional and pivotal roles in enamel development and is particularly needed for ameloblast differentiation and enamel matrix formation.

## Author contributions

Jinxuan Zheng, Rongcheng Yu, Yiqi Tang and Sihui Su: conceptualization, investigation, methodology, data curation, formal analysis, writing-original draft, writing-review & editing, and funding acquisition; Sainan Wang, Chenxi Liao, Xuecong Li, Jiabin Liao, Dongsheng Yu, Tingting Ai, Wei Zhao, and Vicky Yau: methodology, investigation, writing-review & editing; Chufeng Liu, Liping Wu, and Yang Cao: conceptualization, formal analysis, data curation, writing-review & editing, project administration, and funding acquisition. All authors gave final approval and agreed to be accountable for all aspects of the work.

## Funding

This work was supported by grants from the 10.13039/501100001809National Natural Science Foundation of China (No. 81900958, 82170987, 82073378, 81974146, 82101053), the 10.13039/501100003453Natural Science Foundation of Guangdong Province, China (No. 2020A1515010059, 2021A1515012535), Sun Yat-Sen University Clinical Research 5010 Program (No. 2023009), Science and Technology Planning Project of Guangzhou, China (No. 2023A04J2148), and Open Funding of Guangdong Provincial Key Laboratory of Stomatology (China) (No. KF2021120104).

## Conflict of interests

The authors declare no conflict of interests.
